# A Review of the Machining Mechanisms in Field-Assisted Cutting of Brittle Materials

**DOI:** 10.3390/mi17030361

**Published:** 2026-03-15

**Authors:** Xuexiang Sheng, Zhanchen Zhu, Changlin Liu

**Affiliations:** 1 Engineering and Technology Innovation Center, Shandong Harbor Engineering Group Co., Ltd., Rizhao 276800, China; 18063309392@163.com; 2 State Key Laboratory of Ultra-Precision Machining Technology, Department of Industrial and Systems Engineering, The Hong Kong Polytechnic University, Hong Kong 999077, China; 3 School of Mechanical Science and Engineering, Huazhong University of Science and Technology, Wuhan 430074, China

**Keywords:** ultra-precision machining, field-assisted cutting, material removal mechanism, molecular dynamics simulation

## Abstract

Brittle materials such as single crystals, polycrystalline ceramics, and amorphous glass are indispensable in modern industry. Driven by improvements in equipment performance, the required fabrication precision for optical elements and devices has reached nanoscale and is steadily advancing toward atomic level. Despite their outstanding physical and chemical properties, fabricating a defect-free surface with nanometer-level roughness on brittle materials is challenging due to microcracking, brittle fracture and severe tool wear. In recent years, field-assisted cutting has emerged to overcome the bottleneck in ultra-precision cutting of brittle materials. This review summarizes investigations of material removal mechanisms of brittle materials in ultra-precision cutting and surveys representative field-assisted cutting technologies—including laser, vibration, magnetic field, and ion implantation assisted cutting—highlighting how these fields broaden ductile-regime machining and suppress the machining-induced defects. This review further discusses the emerging multi-field coupling strategies and outlines future research directions in machining mechanisms to enable high-efficiency, low-damage, and high-consistency manufacturing of brittle materials.

## 1. Introduction

Owing to the superior optical, electrical, and thermal properties, brittle materials have been widely utilized in the manufacturing of critical components in precision optics, space exploration, and biomedical engineering. Driven by the demand to improve the performance of the devices, the required manufacturing precision for brittle components has advanced to nanoscale and is steadily progressing toward atomic level. However, due to the inherent brittle nature of these materials, surface defects, subsurface damage, and rapid tool wear are often inevitable during machining. These issues have emerged as formidable bottlenecks that impede the achievement of nanometer-level surface quality of brittle materials. In recent years, researchers have demonstrated that brittle materials can be machined in ductile mode via ultra-precision machining (UPM) technologies [1]. By reducing the material removal thickness into nanoscale, the machined surface quality can be significantly improved with less generation of surface defects. Nevertheless, as the material removal thickness decreases to nanoscale, the microstructure of materials and specific machining conditions, such as crystal orientation, pre-existing defects, loading rate, and machining temperature, exert a profound influence on the removal process, rendering the underlying material removal mechanisms highly complex. Furthermore, the maximum material removal thickness for ductile mode removal is quite small, making it difficult to realize large-scale industrial manufacturing.

In recent years, field-assisted cutting has become a focus of academic research in UPM. Its central idea is to introduce controllable external energy fields (such as laser heating, ultrasonic tool vibration, magnetic fields, etc.) on top of conventional cutting processes to actively regulate the local mechanical response of the material or the removal mode of the machining process. This technology can effectively broaden the conditions for ductile-regime removal of brittle materials, reduce the cutting forces, suppress surface and subsurface defects, and ultimately improve the machined surface quality and machining efficiency. The application of field-assisted cutting in UPM has achieved great success in various brittle materials. Furthermore, to amplify the effects of applied assistive fields, researchers have coupled the energy fields and explored the mechanisms in multi-field assisted cutting. This approach provides a novel technical route for enhancing the machining quality of brittle materials. In field-assisted cutting, the introduced energy field alters both the intrinsic properties of materials and the tool–workpiece interaction. Consequently, the underlying machining mechanisms differ fundamentally from those of conventional processes, making this a prominent research hotspot in the current field of UPM.

**Figure 1:**
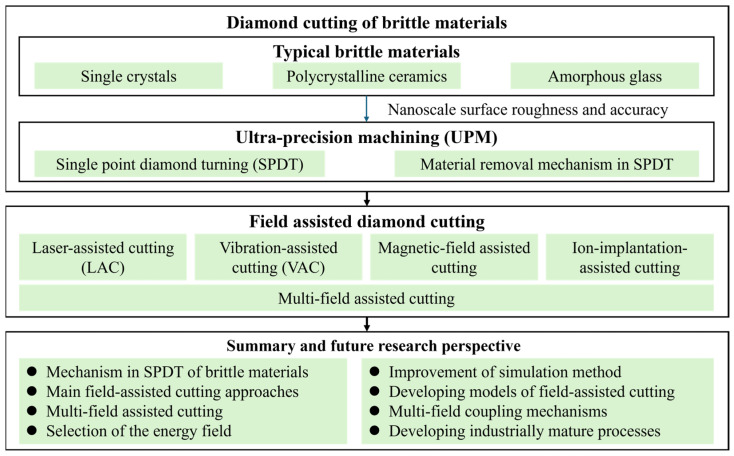
Structural diagram of the review.

This review focuses on the research trajectory and key issues in field-assisted cutting of brittle materials, as shown in Figure 1. In Section 2, we provided an overview of representative brittle materials and their material removal mechanisms during UPM. We then discussed the development and machining mechanisms of representative field-assisted cutting technologies in Section 3. Based on a synthesis and analysis of the current state of the art, we summarized the current research in Section 4 and further discussed challenges and research directions in Section 5. Through these contents, this paper aims to provide a systematic reference for academic investigation and engineering implementation of field-assisted cutting in the high-quality manufacturing of brittle materials.

## 2. Diamond Cutting of Brittle Materials

### 2.1. Typical Brittle Materials

**Figure 2:**
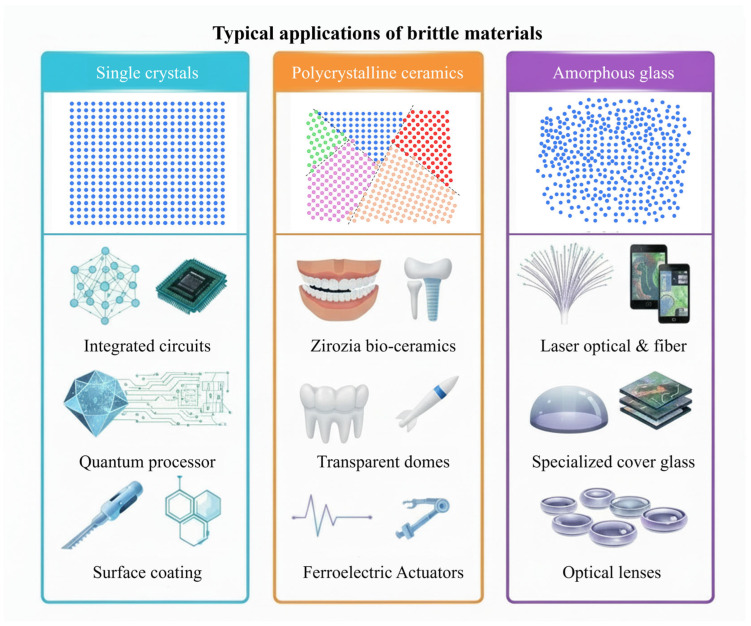
Typical applications of brittle materials.

Brittle materials refer to a class of materials that fracture with little deformation when subjected to stress (the critical strain normally being below 5% [2]). Brittle materials usually exhibit good wear resistance and corrosion resistance, high stiffness, and excellent semiconductor properties, making them key components in advancing modern technology, such as integrated circuits, precision instruments, and space optics [3,4,5]. Most of the inorganic non-metallic materials are brittle materials, including single crystals, polycrystalline ceramics, and amorphous glass, as illustrated in Figure 2.

**(a) Single crystals**

Single crystals such as silicon, silicon carbide, and quartz are materials in which atoms are linked throughout the entire structure by strong, directional covalent bonds, forming a continuous 3D (or sometimes layered) bonding network rather than discrete molecules. Because of this bonding, single crystals exhibit unique properties including high melting points, hardness and brittleness [6,7]. The arrangement of atoms in single crystals plays a crucial role in determining their stability and overall characteristics, making them distinct in the context of molecular interactions and crystal formations.

Single crystals are widely used in the fields of microelectronics, precision optics, and advanced manufacturing [8]. For example, as a highly ordered form of atoms, single-crystal silicon has uniform electrical and mechanical properties, making it the most important substrate material in modern electronics such as integrated circuits [9], power devices [10], and micro-electro-mechanical systems (MEMS) [11]. Owing to its advantages in optical and mechanical properties, single-crystal silicon is widely used for infrared windows [12], X-ray mirrors [13], and components in harsh environment systems [14]. Furthermore, as a representative third-generation semiconductor, single-crystal silicon carbide is an important material in high-power and high-temperature electronics as well as optical components under harsh thermal and mechanical conditions [15]. Moreover, very hard covalent crystals such as diamond and cubic boron nitride are essential materials in abrasives [16], cutting tools [17], and protective coatings [18]. Diamond is also a prominent candidate for next-generation semiconductor devices owing to its extraordinary combination of electronic, thermal, mechanical, and quantum properties [19].

**(** **b** **) Polycrystalline ceramics**

Ceramic materials are a class of materials composed primarily of inorganic non-metallic compounds, typically manufactured through powder batching, forming, and high-temperature sintering. Polycrystalline ceramic is a composite whose properties reflect the properties of its grains and grain boundaries. Typical polycrystalline ceramics include alumina, zirconia, silicon nitride, and aluminum nitride. Because of the strong ionic and covalent bonding, polycrystalline ceramics generally exhibit high hardness and wear resistance, high-temperature capability and corrosion resistance, and good electrical insulation and dielectric properties.

Polycrystalline ceramics have widespread structural and mechanical applications. Owing to the high specific stiffness and dimensional stability, reaction-bonded silicon carbide is a promising material for large-aperture mirrors used in the telescope community worldwide [20]. Bio-ceramics such as alumina and zirconia are mainly used in orthopedic and dental repair due to their compatibility with the physiological environment, desired mechanical strength and wear resistance [21]. Alumina is also a key optical material used in transparent windows and domes for harsh environments such as infrared missile domes, high-pressure viewports, optical coatings, and protective sensor windows [22]. Furthermore, ferroelectric ceramics such as lead zirconate titanate, barium titanate and lead lanthanum zirconium titanate are widely used in electrocaloric cooling, electromechanical actuators, and transducers renowned for their desired properties including exceptional electrocaloric effects and high piezoelectric coefficients [23,24].

**(** **c** **) Amorphous glass**

Amorphous glass is an inorganic, non-metallic solid that has a non-crystalline atomic structure. It is typically produced by melting raw materials and cooling the melt fast enough to avoid crystallization, forming a rigid, strongly bonded network [25]. Glass is a good electrical insulator with relatively high chemical stability, hardness and brittleness. Unlike crystalline materials, glass has no mobile dislocations to blunt crack tips, and small surface flaws such as scratches or microcracks could create strong stress concentrations during deformation. Under tensile or bending loading, these cracks could propagate rapidly, causing sudden fracture with little or no yielding. Glass is widely used in optical systems owing to its high transparency, homogeneity, and precise controllability in refractive index and dispersion [26]. In imaging systems, optical glasses are widely used to manufacture lenses (camera, microscope, telescope, eyeglasses) and prisms that refract and shape light while minimizing aberrations [27]. In laser and photonics systems, glass serves as windows, beam splitters, and mirrors. It is also the core material for optical fibers used in telecommunications [28] and hemispherical resonator gyroscopes in navigation systems [29]. Specialized glasses are additionally used for UV optics, infrared-transmitting optics, and protective cover plates for displays and detectors where optical clarity and surface quality are critical [30].

Driven by the demand for improving device performance in these fields, precision components are facing increasingly stringent requirements for machining quality and material properties [31,32]. High-end equipment often demands nanometer-level surface roughness and extremely low subsurface machining damage [33,34]. However, brittle materials face significant difficulty in fabricating high-quality surfaces with nanoscale surface roughness and low subsurface damage. During the machining process, cracks tend to initiate and surface defects tend to propagate rapidly, leading to sudden fracture with little to no deformation in the workpiece material [35,36]. Furthermore, rapid cutting tool wear, fluctuations in machining forces, pronounced thermal effects, and significant residual stresses further raise the threshold for achieving nanometer-level surface quality. Therefore, achieving high-precision, low-damage machining of these brittle materials has become a major research focus in the field of advanced manufacturing.

### 2.2. Ultra-Precision Cutting Technology

To meet the machining quality requirements of components used in high-end equipment, UPM technologies—including ultra-precision scratching [37,38], cutting [39], grinding [40,41], and polishing [42,43]—have gradually become indispensable methods in advanced manufacturing. Among these technologies, ultra-precision cutting represented by single-point diamond turning (SPDT) has become the preferred technique for UPM of complex structures owing to its advantages of high flexibility, high accuracy, and high machining efficiency [44]. During ultra-precision cutting, the workpiece is fixed on a high-precision air/hydrostatic spindle and rotated at a controlled speed [45]. A diamond tool is mounted on ultra-precise linear slides and moved under CNC control. During machining, the spindle rotates to provide the primary cutting motion while the tool follows a programmed tool path to generate feed and the desired surface geometry. Relying on nanometer-scale positioning, excellent spindle error motion control, high machine stiffness, and a sharp cutting edge, optical-quality surfaces with high form accuracy and low surface roughness can be achieved. In its early stage of development, ultra-precision cutting was mainly applied to machine ductile metals such as aluminum and copper. With the advancement of space optics and defense systems, the demand has grown rapidly for ultra-precision optical components made from brittle materials such as single-crystal silicon and silicon carbide. This demand has driven the expansion of ultra-precision cutting into machining of brittle materials.

### 2.3. Removal Mechanisms of Brittle Materials

**Figure 3:**
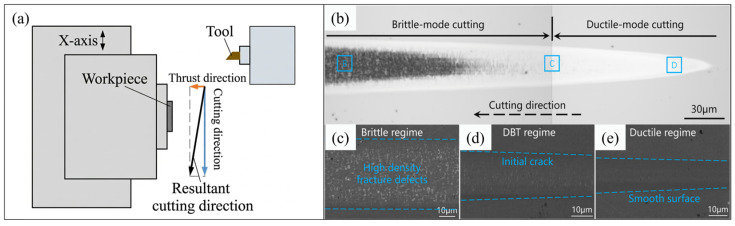
Determination of the removal characteristics by plunge-cutting experiments. (**a**) Illustration of the plunge-cutting experiments. (**b**) Surface morphology of the machined surface from the plunge-cutting experiment, and (**c**–**e**) micrograph of the surface from brittle, transition, and ductile mode machining [53].

**Figure 4:**
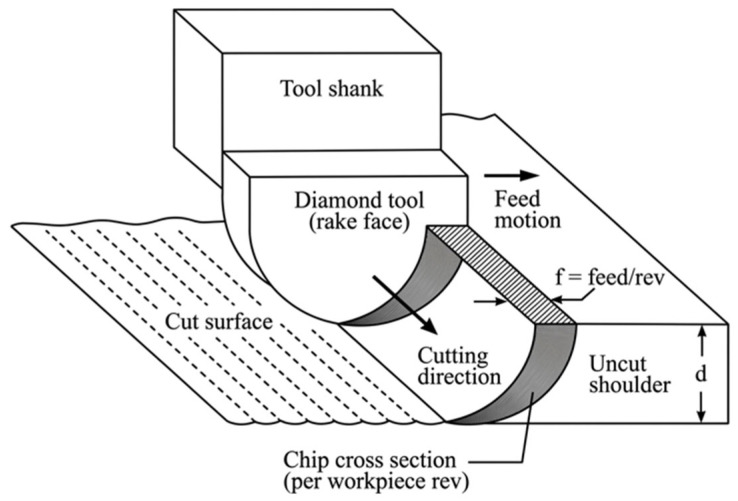
Geometric feed model for ultra-precision cutting.

Unlike ductile metals, brittle materials have high hardness and low plasticity. Surface defects such as micro-chipping and cracking are generally inevitable during the machining process [46,47]. Conventional processes therefore struggle to produce surfaces with high accuracy and low subsurface damage. Based on extensive cutting experiments, scholars have revealed that there are three typical characteristics of material removal when brittle materials are cut by decreasing the removal thickness: brittle removal, brittle–ductile transition, and ductile removal, which can be clearly identified by plunge-cutting experiments, as illustrated in Figure 3a. In the brittle removal stage, materials are mainly removed by cracks, chipping, and fractures with irregular plastic deformation, generating surfaces with severe defects and subsurface damage, as shown in Figure 3c. As the removal thickness decreases, the material removal mechanism could gradually transit into ductile mode, making it possible to fabricate high-quality surfaces of brittle materials, as shown in Figure 3d,e. In 1989, Puttick et al. [48] claimed the existence of a critical material removal thickness during SPDT below which the brittle material can be removed by stable plastic deformation, leaving a crack-free machined surface. In the 1990s, Blake and Scattergood [49] introduced the concept of critical material removal thickness for hard and brittle materials through cutting experiments. They proposed a feed model to determine the geometric relationship between the tool and the workpiece and the ductile–brittle transition in SPDT, as shown in Figure 4. Based on this model, the ductile–brittle transition is closely related to tool geometry and cutting parameters, which is further confirmed by Cai et al. [50]. Yan et al. [51] pointed out that the high compressive stress introduced by decreasing the tool rake angle or material removal thickness is an important reason for ductile machining of hard and brittle materials. Afterwards, the ductile machinability of various brittle materials has been confirmed in SPDT [52].

In macroscale cutting of metallic materials, the material removal thickness is typically on the micrometer scale, which is much larger than the tool edge radius. Therefore, the material removal process is mostly described using the classical shear model without considering the size effect of the cutting tool edge [54]. In the ductile removal stage of ultra-precision cutting, the material removal thickness decreases to the nanoscale, which is in the same order of magnitude as the tool edge radius. Therefore, the size effect of the cutting tool edge has a pronounced influence on the material removal behavior. Due to the difficulty in direct observation of material deformation at the nanoscale, numerical simulation has become a preferred method to explore the mechanisms during UPM. Scholars have conducted extensive numerical investigations to reveal the material removal behavior, subsurface structure evolution, and cutting tool wear in UPM via finite element (FE) simulation [55], discrete element method (DEM) [56], smoothed particle hydrodynamics (SPH) [57], and molecular dynamics (MD) simulation [58]. In particular, MD simulation, which is based on first-principles calculations or empirical interatomic potentials, has been widely used to describe the material deformation behavior in UPM owing to its advantages in simulating plastic deformation behavior such as dislocation propagation and phase transition [59,60,61]. In the 1980s, Lawrence Livermore National Laboratory first applied MD simulation to investigate the material deformation mechanism of single-crystal copper. In the early stages, such studies were limited in the size of the simulation system due to computational constraints. Nowadays, with the development of computer technology and interatomic potentials, MD simulation has gradually become a primary approach for studying the nanoscale cutting mechanisms of brittle materials including single crystals [62], polycrystalline ceramics [63], and amorphous glass [64].

**Figure 5:**
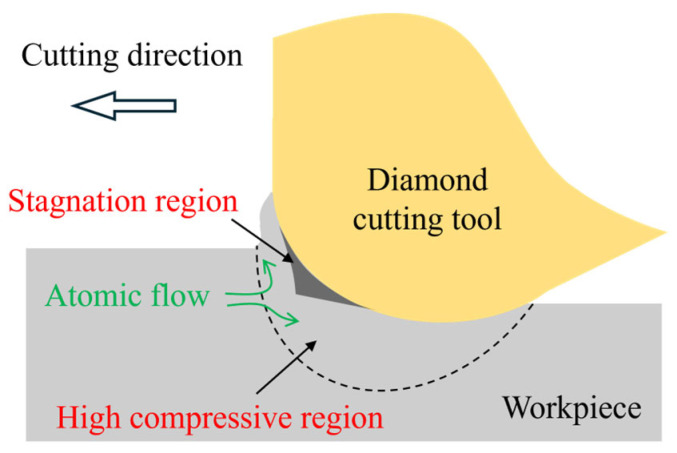
Schematic illustration of the material removal behavior during ultra-precision cutting.

For single crystals, phase transition and dislocation slip are two dominant mechanisms of plastic deformation. During ultra-precision cutting, the high compressive stress introduced by the tool edge causes localized structure evolution in the tool–workpiece contact region. Fang et al. [65] first proposed that the dominant material removal mechanism in ultra-precision cutting of single-crystal silicon is extrusion, as shown in Figure 5. They suggested that the compressive stress near the tool edge causes high-pressure phase transformation (HPPT) of the cubic diamond phase into the high-pressure phase (e.g., the Si-II phase) in the deformation zone [66], thereby enabling the extrusion of the deformed material during cutting [67]. Another feature of extrusion is the existence of a so-called stagnation region ahead of the tool edge [68], where the workpiece atoms are nearly stationary relative to the cutting tool during the cutting process. Atoms above the stagnation region are piled upward to form chips and ridges through extrusion, while atoms below the stagnation region are compressed downward into the workpiece. After cutting, the compressed material undergoes further transformation into an amorphous phase, forming an amorphous layer that remains on the machined surface [69].

**Figure 6:**
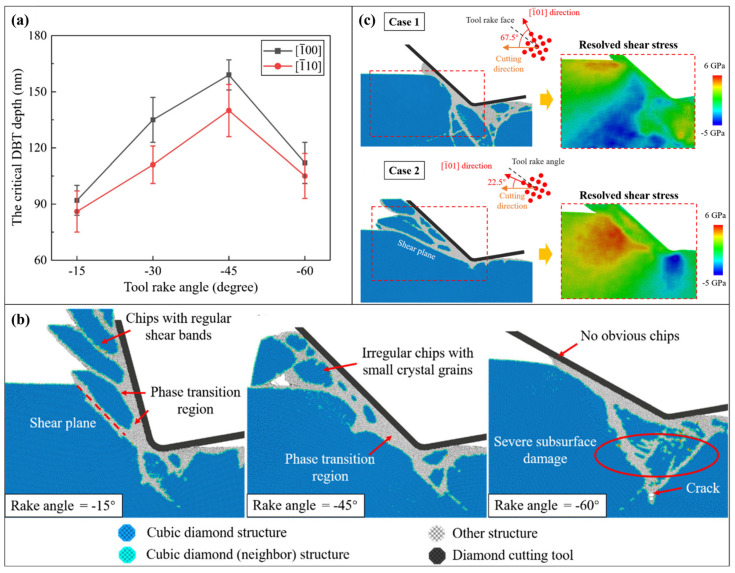
The material removal behavior of single-crystal silicon. (**a**) Influence of tool rake angle on the critical depth of cut for ductile removal. (**b**,**c**) Material removal behavior under different tool rake angles and crystal orientations of workpieces [74].

When the material removal thickness increases, the removal mechanism of single crystals shows new features other than extrusion. Kausala and Zhang [70] found that for single-crystal silicon, nanotwins can be observed in the subsurface workpiece when the scratch depth exceeds 1 nm, which indicates the existence of slip motion during cutting. When the material removal thickness further increases, the deformation mechanism shows some features similar to those of macroscale cutting. Based on an MD cutting simulation with a removal thickness increasing to 20 nm, Liu et al. [71] suggested that the material removal mechanism shifts from extrusion to shearing as the cutting tool edge radius decreases. This transition occurs when the tool edge radius is approximately equal to the material removal thickness. To quantitatively characterize this relationship, Yan et al. [72] proposed the relative tool sharpness (RTS), which is defined as the ratio between tool edge radius and material removal thickness. They pointed out that as RTS increases, the dominant removal mechanism of single-crystal silicon transitions from rubbing (0 < RTS < 0.1) to ploughing (0.1 < RTS < 0.25), then to extrusion (0.25 < RTS < 0.5), and finally to shearing (0.5 < RTS) [72]. This transition was further observed for other single crystals such as silicon carbide [73]. In addition to tool edge radius, Liu et al. [74] pointed out that the tool rake angle and crystal orientation of workpieces have a significant influence on the dominant material removal mechanism of single crystals, as illustrated in Figure 6.

**Figure 7:**
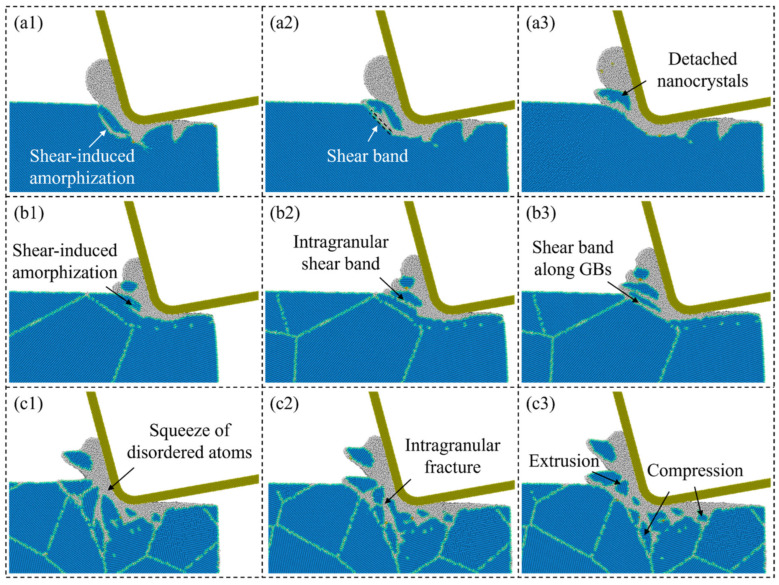
Snapshot of the material removal behavior during cutting on: (**a1**–**a3**) a single-crystal workpiece; (**b1**–**b3**,**c1**–**c3**) polycrystal workpieces with different grain sizes [82].

Unlike single crystals, polycrystalline ceramics present complex material removal mechanisms due to the randomly distributed crystal grains and grain/phase boundaries [75]. Multiple material deformation and removal mechanisms coexist and interact during the cutting process, and their dominance depends strongly on the machining conditions and microstructural characteristics. The random arrangement of grains and boundaries indicates that polycrystalline ceramics can be effectively modeled as an assemblage of bonded discrete particles, making numerical approaches such as DEM and MD particularly suitable for studying the deformation features during machining [76,77]. At small material removal thickness, plastic deformation can occur locally, and the material removal mechanism is similar to that of single crystals [78]. Nevertheless, because of the discrete bonding between grains, friction between the tool and the workpiece could introduce micro-cracks near the grain/phase boundaries [79]. Meanwhile, breakage of intergranular bonds leads to intergranular fracture and grain boundary separation [80], making it difficult to fabricate a defect-free surface on polycrystalline ceramics even when the single-crystal components are removed in ductile mode [81]. In practical machining, polycrystalline ceramics usually exhibit mixed ductile–brittle material removal features. Smooth regions produced by plastic deformation or amorphization coexist with fractured regions caused by intergranular cracking [82], as shown in Figure 7. With the increase in material removal thickness, crack propagation could appear inside the grains, causing brittle mode removal that is similar to that of single crystals, coupled with intergranular fracture and grain boundary separation.

**Figure 8:**
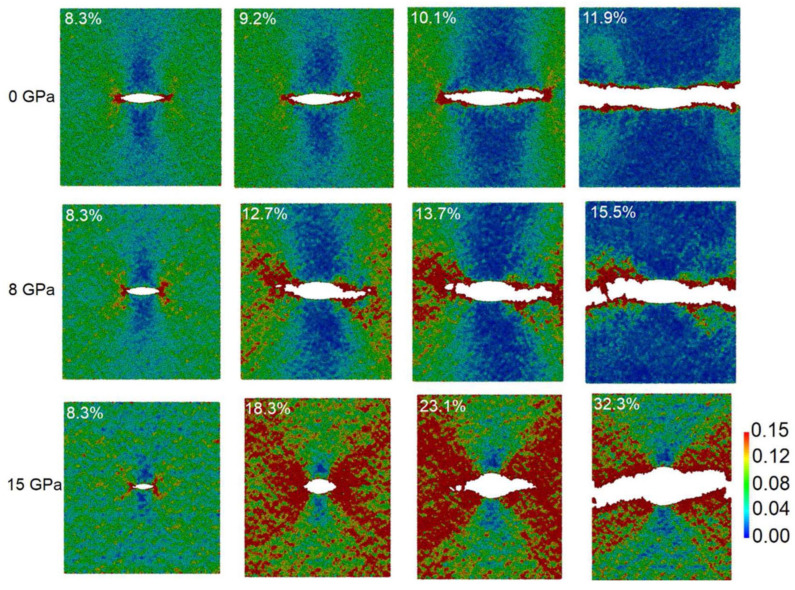
Local shear strain mapping of a V-crack tension test under different stresses of fused silica glass via MD simulation [89].

For amorphous glass, the machining mechanism is distinct from that in crystalline materials, since atoms in glass are not arranged in periodic lattices, which fundamentally limits conventional plastic deformation mechanisms such as dislocation motion and phase transition. The three-dimensional network formed by randomly arranged and interconnected atoms contains massive atomic-scale voids and dangling bonds, resulting in a relatively low packing density of atoms [83]. During UPM, amorphous glass undergoes localized plastic deformation through densification and shear flow of the glass network at small material removal thickness [84], which contributes to ductile-mode material removal. Taking fused silica as an example, bonds between silicon and oxygen atoms undergo twisting and bending to fill the voids during machining, leading to variations in nanoscale structure such as n-membered rings [64] and densification [85]. Furthermore, fused silica exhibits localized plastic flow due to surface friction and shear stress under compression and shear deformation (shown in Figure 8), which contributes to pile-up and material removal during the machining process [86,87]. When the material removal thickness increases, median and radial cracks may initiate due to stress concentration, which is an important reason for the transition to micro-brittle fracture and chipping [88].

In summary, the ultra-precision cutting technologies represented by SPDT have enabled ductile machining to fabricate defect-free surfaces of a variety of brittle materials. Scholars have conducted extensive in-depth investigations into the underlying mechanisms of the machining process through experiments and numerical approaches such as MD. It is worth mentioning that due to limitations in the accuracy of interatomic potential and computational capacity, discrepancies still exist between MD simulations and experiments in terms of simulation scale and machining parameters. Furthermore, tool wear and subsurface damage caused by the inherent hardness and brittleness of materials, as well as the relatively low ductile removal thickness that results in limited machining efficiency, remain critical bottlenecks restricting improvements in quality and efficiency of UPM for optical components. As the research into the nanometric cutting mechanism of brittle materials continues to deepen, enhancing ductile machinability, suppressing cutting tool wear, and reducing subsurface damage have become the key issues in improving the manufacturing capability of brittle optical components.

## 3. Field-Assisted Cutting Technology

**Figure 9:**
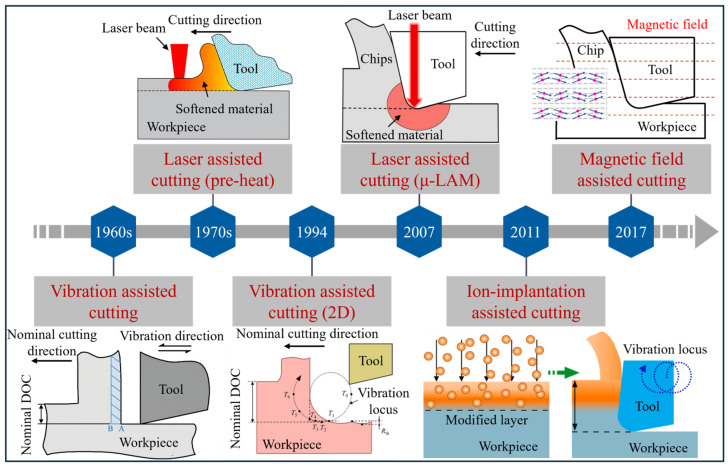
Development of typical field-assisted cutting technologies.

At present, it is difficult to further improve the machining quality solely by adjusting the machine tools and process parameters. In recent years, field-assisted cutting technologies, such as laser heating, tool vibration, and magnetic field, have provided new approaches to enhance the machinability of brittle materials and attracted increasing attention from the academic community. Figure 9 illustrates the development of typical field-assisted cutting technologies [90,91,92]. This section introduces the development and mechanisms of various field-assisted cutting technologies commonly used in UPM of brittle materials.

### 3.1. Laser-Assisted Cutting

**Figure 10:**
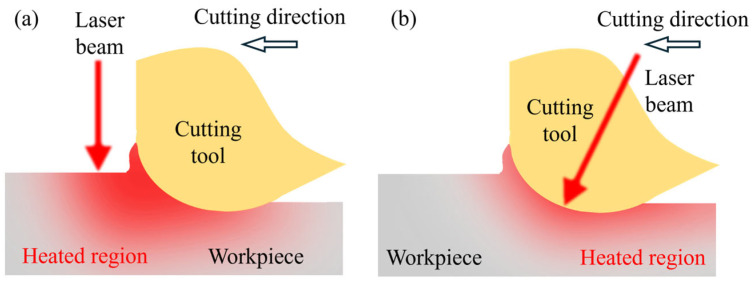
Schematics of the heating strategy in LAC. (**a**) Pre-heat LAC. (**b**) In situ LAC.

Since the hardness of materials can be decreased by raising the temperature, thermal field can be used as an auxiliary approach to improve the machinability of hard and brittle materials [93]. In the 1950s, thermal assistance had already emerged in the machining of hard-to-machine materials to achieve better machining performance. It has been proven that with thermal assistance, higher material removal rate [94], better surface quality [95] and lower cutting tool wear [96] can be achieved for hard and brittle materials, as shown in Table 1. During the machining process, a highly controllable heat source is crucial to control the thermally influenced region. Common heat sources in practical applications include plasma [97], laser beams [98], gas heating [99], and induction heating [100]. With the advancement of laser technology, laser-assisted cutting (LAC) has become a research hotspot in the field of thermal-assisted machining owing to its advantages of high positioning accuracy and energy density of the thermal field. In the 21st century, in situ laser-assisted diamond turning was proposed by Shayan et al. [101]. By adjusting a laser beam that passes across the diamond tool edge, precise control of the laser energy can be achieved, as illustrated in Figure 10b. This heating method allows precise control of the heat distribution within the workpiece, avoiding the overheating caused by conventional laser heating (shown in Figure 10a) and thereby effectively suppressing thermal expansion and thermal damage of the workpiece [102]. At present, this technique has been successfully applied to the ultra-precision cutting of various brittle materials such as single-crystal silicon [103], fused silica [104], silicon carbide [105], sapphire [106], and silicon nitride ceramic [107].

**Figure 11:**
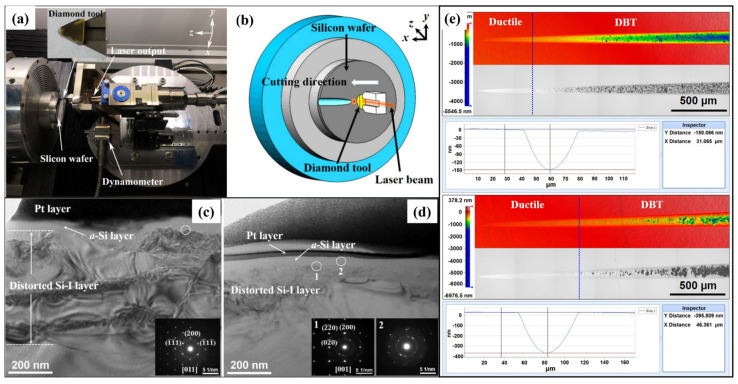
LAC for single-crystal Si. (**a**,**b**) Experimental setup of LAC. (**c**,**d**) TEM images of the subsurface workpiece in ductile cutting regions. (**e**) Improvement of the critical depth of cut by applying LAC [108].

**Figure 12:**
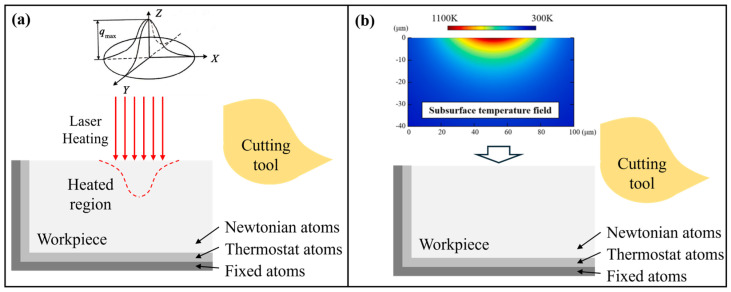
MD model for LAC where (**a**) the laser heating is simulated by adding a specific heating region and (**b**) the entire workpiece is heated to the desired temperature according to FE simulation of the temperature distribution [91].

During LAC, the machining mechanism differs from that of conventional cutting since the material properties and deformation features are significantly affected by temperature [110,112]. For brittle materials, the cutting forces and internal stress in the deformation zone [113] decrease significantly with increasing temperature due to the thermal softening effect, contributing to better machining performance and lower abrasive tool wear [114]. By plunge-cutting experiments with an in-situ laser heating module shown in Figure 11a, Chen et al. [108] confirmed the improvement of the machinability of single-crystal silicon by LAC, as shown in Figure 11b–e. Their results indicate that the critical depth of cut for single-crystal silicon can be improved from 150 nm to 395 nm with the assistance of laser heating. Furthermore, the phase transition behavior and dislocation motion change markedly under high-temperature conditions, causing material removal behavior that is distinct from that of conventional cutting. Because the material is removed at the nanoscale with high strain rate and temperature, MD simulation is a suitable tool for exploring the removal mechanism in LAC. In MD simulation, the laser heating process can be simulated either by a specific heating region [115,116] or heating the whole workpiece to a desired temperature obtained by FE simulation [91], as shown in Figure 12. Based on previous research, atomic flow in the deformed region is promoted when the temperature rises, which dissipates more strain energy and suppresses the elastic recovery on the machined surface [91]. For single-crystal silicon, the extrusion removal is suppressed since the internal compressive stress is decreased and fewer amorphous atoms are generated [115]. Furthermore, Chen et al. [108] found that the amorphous phase can be generated directly from the cubic diamond phase via lattice collapse without the formation of intermediate high-pressure phases. The newly formed amorphous phase partially crystallizes during cutting and transforms into the Si-III and Si-XII phases under annealing. In addition to phase transition, they suggested that laser heating promotes dislocation activity, thereby greatly enhancing cleavage and shear removal at high temperatures [117,118]. Similar results were reported by Meng et al. [119], and they pointed out that increasing the machining temperature is beneficial for triggering the nucleation and extension of dislocations in the glide slip system, which is sensitive to high temperature.

For crystalline materials, laser heating introduces crystallization of the disordered atoms beneath the machined surface, which is beneficial for reducing the subsurface damage generated during machining. As early as 1979, researchers found that the crystallization of amorphous silicon is closely related to temperature [120]. During ultra-precision cutting, the disordered phases could recrystallize into a crystalline structure with increasing temperature [121]. For silicon, this process can be promoted by increasing compressive stress during subsequent processing, since the cubic diamond phase has a close-packed atomic structure and higher density than the amorphous phase [122]. However, recrystallization into other phases is usually inevitable during LAC. Langana et al. [123] found that excessive heating power introduces multiple crystalline phases on the machined surface, accompanied by undesired residual stress. From MD simulation, Chen et al. [124] reported the existence of recrystallization into a hexagonal structure of single-crystal silicon during annealing of the machined workpiece at high temperature. For polycrystalline ceramics, different crystallization features can be observed due to the existence of grain boundaries and mismatch in material properties between phases. Liu et al. [125] revealed that the recrystallization of silicon is more apparent than that of silicon carbide during LAC of reaction-bonded silicon carbide.

The above-mentioned studies have demonstrated the advantages of LAC in machining brittle materials, as well as the laser-induced changes in material removal behavior and subsurface damage mechanisms. However, how laser heating influences the subsurface structure is mainly investigated by MD simulation and post-process observation, which lacks convincing evidence of the dynamic evolution of microstructure during machining. Furthermore, regardless of the benefit in tuning the material properties of the workpiece, laser heating could reduce the hardness and increase the chemical reactivity of the cutting tool [126], which may lead to severe chemical tool wear [127]. Therefore, more detailed investigation of the thermal softening effect should be conducted, and LAC must strike a balance between workpiece softening and tool softening to achieve optimal machining quality.

### 3.2. Vibration-Assisted Cutting

**Figure 13:**
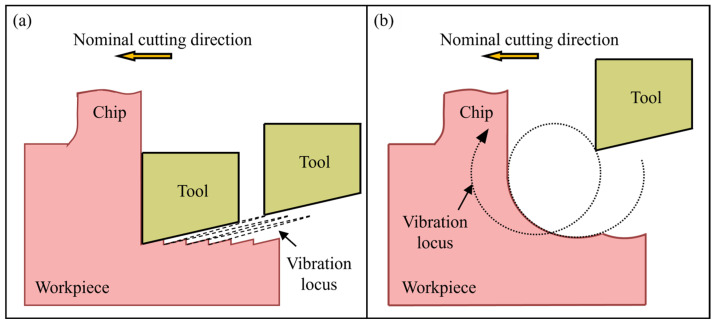
Schematic illustration of (**a**) LVC and (**b**) EVC.

Vibration-assisted cutting (VAC) has been applied in the manufacturing industry since the 1960s. In its early applications, the cutting tool undergoes one-dimensional linear vibration along the nominal cutting direction, which is also known as linear vibration cutting (LVC) [128], as illustrated in Figure 13a. Compared with conventional cutting, LVC effectively reduces the cutting forces [129], mitigates process instability [130], and extends cutting tool life [131]. However, the tool’s linear vibration tends to imprint vibration marks on the machined surface, thereby degrading the surface quality. To address this issue, Shamoto and Moriwaki proposed two-dimensional elliptical vibration cutting (EVC) [132], in which the cutting tool vibrates in two dimensions within the plane defined by the nominal cutting direction and the nominal depth of cut direction, as illustrated in Figure 13b. During EVC, the instantaneous material removal thickness is much smaller than the nominal depth of cut, and the cutting force acting on the cutting tool is markedly lower than that in conventional cutting. Furthermore, owing to the periodic contact and separation between the tool and the workpiece, most of the heat generated during cutting is removed by air and cutting fluid. At present, EVC has been successfully applied in UPM of a wide range of brittle materials, including single-crystal silicon [133], sintered silicon carbide [134], tungsten carbide [135], calcium fluoride [136], and composite materials [137].

During EVC, the high-frequency tool vibration introduces motion along the depth of the cut direction, rendering the machining mechanism more complex than that in conventional cutting. Scholars have investigated the mechanisms of surface formation and material removal of various brittle materials in EVC and demonstrated the significance of tool vibration on cutting performance [138,139]. It is revealed that the tool vibration-introduced reduction in material load is responsible for the decrease in cutting forces [140] and internal stress [141]. Furthermore, the variation in transient cutting tool motion has a significant influence on the machining anisotropy when cutting single crystals. Chen et al. [142] suggested that the tool vibration dynamically redistributes and rebalances the existing anisotropy among the applied forces, thereby leading to unique machining characteristics, including homogeneous deformation. Moreover, the tool piling-up motion facilitates the upward motion of materials, which tends to enhance the atomic flow and increase the material removal rate [143]. The tool vibration-introduced upward motion causes an undesired increase in tensile stress during the cutting process, which causes tearing of brittle materials such as single-crystal silicon [144]. Therefore, investigation into the mechanisms and optimization of vibration parameters such as nominal cutting speed, vibration amplitude, and vibration frequency constitutes an important research direction in VAC.

The nominal cutting speed is considered one of the most essential parameters in EVC [145]. It is revealed that the decrease in nominal cutting speed causes an obvious reduction in material load, which is beneficial for suppressing tool wear and extending tool life [146]. Meanwhile, nominal cutting speed is an important parameter in determining the surface quality since it determines the theoretical surface roughness [147]. Nath and Rahman [148] found that a lower nominal cutting speed can achieve a better surface finish, which is further confirmed by Huang et al. [149]. In addition to the nominal cutting speed, the vibration amplitude and frequency also have a significant influence on the machining performance. From the results of Dai et al. [150], a lower vibration frequency and a smaller amplitude ratio can increase the material removal rate when machining single-crystal silicon. Liu et al. [151] pointed out that the vibration amplitude affects the deformed region by influencing the piling-up motion of the workpiece material. Their simulation results indicate the existence of an optimal ratio of vibration amplitude in cutting single-crystal silicon, which has been verified by experiments [144]. For the material removal process, it is suggested that the shear angle increases and the chip thickness decreases as the frequency or amplitude increases [152], which promotes shear removal of workpiece material. Furthermore, Li et al. [153] suggested that the increase in normal amplitude enhances discontinuous processing while the increase in normal vibration amplitude promotes the elastic recovery on the machined surface, which decreases the material removal efficiency.

**Figure 14:**
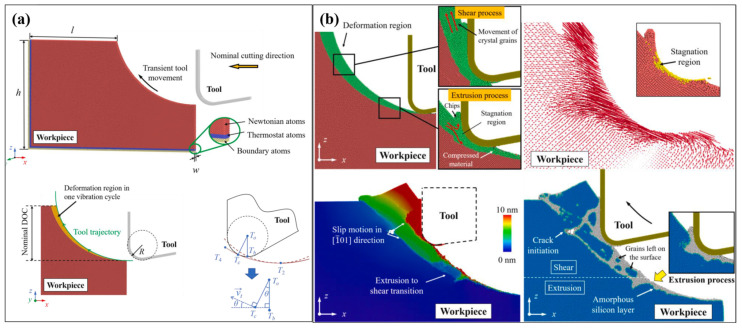
EVC model for simulating the contact stage in one vibration cycle [154]. (**a**) Illustration of the cutting model. (**b**) Transition of the material removal mechanism.

At present, MD simulation is the dominant method for the explanation of the transient material removal mechanism in EVC. Due to limitations in model size, MD simulations of UPM typically employ models with material removal thickness ranging from several to tens of nanometers. Because the tool vibration in EVC leads to an instantaneous material removal thickness much smaller than the nominal depth of cut, prior MD models for EVC simulation commonly assume an instantaneous material removal thickness of only 1–2 nm [151], which impedes the accurate description of the material removal mechanism. To overcome this problem, Liu et al. [154] proposed a cutting model to simulate the contact stage in one vibration cycle, which effectively enlarged the transient material removal thickness to ~10 nm with a much lower instantaneous tool velocity, as shown in Figure 14a. They revealed that the transient deformation mechanism of single-crystal silicon experiences the transition from extrusion to shearing in one vibration cycle by analyzing the transient material removal process shown in Figure 14b. Based on this model, they investigated the extension of subsurface damage and discussed the variation in resolved shear stress and dislocation propagation of single-crystal silicon during EVC [90]. A main drawback of this model is that it failed to evaluate the machined surface morphology since only one vibration cycle is involved in the simulation. Therefore, establishing a more realistic simulation system is important to fully reveal the surface formation mechanism during EVC.

### 3.3. Magnetic Field-Assisted Cutting

**Figure 15:**
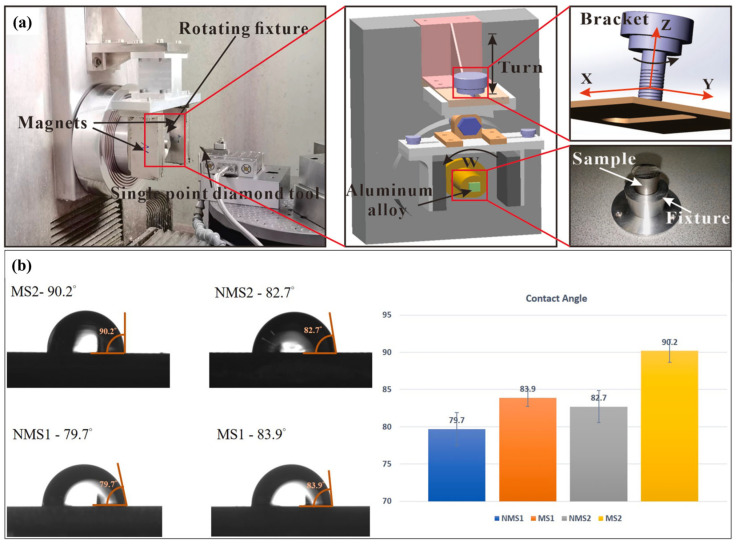
Magnetic field-assisted diamond turning. (**a**) The experiment setup. (**b**) The contact angle measurements of machined micro-structured surfaces [156].

In addition to thermal and vibration assistance, magnetic field-assisted cutting is another field-assisted cutting method, with industrial applications dating back as early as the 1960s. Compared to other energy fields used in UPM, magnetic fields offer several advantages, including low cost, simple operation, and ease of removal. In 2017, Yip and To proposed the first magnetic field-assisted cutting technology [155], aiming to minimize the material swelling effect during ultra-precision cutting of titanium alloys. Their results indicated that the magnetic field effectively increases the material removal rate and machined surface quality in the fabrication of smooth surfaces and micro-structured surfaces [156], as shown in Figure 15. This technique is mainly applied in machining magnetically susceptible materials, and it has achieved good results in the machining of brittle materials such as calcium fluoride [157]. It is revealed that a weak magnetic field introduces magneto-plasticity via the formation of non-singlet electronic states in defected configurations for brittle materials, which indicates that the magnetic field could affect the machining performance of non-magnetic brittle materials [158]. Guo et al. [159] suggested that the application of a magnetic field reduces the internal stress and facilitates the dislocation activity during machining, which is responsible for the enhancement in magneto-plasticity. In addition to the material property, the eddy-current damping effect under a magnetic field can effectively suppress vibrations in the machining system and improve the stability during the cutting process [160], which is advantageous for improving the machined surface quality. Although magnetic field-assisted cutting offers numerous advantages, its current application is restricted to a narrow range of materials due to the material-selective nature of magnetic fields. Furthermore, magnetization can adversely affect the service performance of components, which deteriorates the performance of devices in fields such as aerospace and precision metrology. Therefore, magnetic field-assisted cutting is generally not used, or demagnetization measures are employed after machining to ensure the functional performance of the machined components [161].

### 3.4. Ion Implantation-Assisted Cutting

Ion implantation is a surface-modification technique that alters the physicochemical and biological properties of materials by accelerating high-energy ions and implanting them into the surface. Compared with other doping strategies, ion implantation can introduce almost any element into the target material without additional impurity elements. During the implantation process, the accelerated high-energy ions are implanted into the target material. After multiple collisions, the energy is transferred to target atoms or electrons, causing structural damage that is closely related to the implantation parameters such as ion species, implantation energy, and dose. Owing to the collision-cascade effects, one implanted ion can create tens of thousands of point defects such as vacancies and interstitials in the target material. With increasing implantation dose, defect clusters such as amorphous phases can be generated in materials [162]. These defects can be used to tune the properties of the target material [163] to improve the machinability of brittle materials.

**Figure 16:**
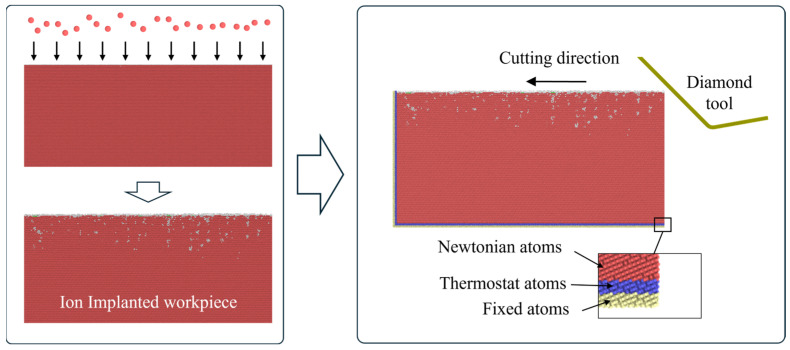
MD simulation of ion implantation-assisted cutting.

The damage structures introduced by ion implantation reduce the hardness and the brittleness of material, making it possible to mitigate brittle fractures and promote plastic deformation during UPM. In 2009, Xu et al. [164] developed a fabrication method combining focused ion beam implantation with gas-assisted etching. They successfully produced a series of micro/nanostructures such as nanogratings, nanoelectrodes, and sinusoidal microstructures. In 2011, ion implantation-assisted cutting was proposed by Fang et al. [165]. In this method, fluorine ions were implanted into single-crystal silicon to form an amorphous layer on the surface, after which ductile-regime material removal could be enabled with the ductile–brittle transition depth significantly increased to 923.566 nm. To reveal the interaction mechanism between high-energy ions and workpiece material, extensive experimental and MD investigations have been conducted owing to their advantages in simulating the structural evolution during ion implantation, as illustrated in Figure 16. Based on three-dimensional MD simulations, Chen et al. [166] proposed that the implantation causes lattice transformation into amorphous phase of single-crystal silicon, which is beneficial for reducing brittleness and fractures during machining. Furthermore, the generated amorphous phase could absorb shear strain energy and make the material more prone to plastic deformation, thereby increasing the critical depth of cut [167]. To obtain the ion distribution after implantation, To et al. [168] established a distribution model of implanted hydrogen ions and visualized the displacement damage induced in the subsurface of single-crystal silicon. These studies validated the effectiveness of ion implantation surface modification in improving the machinability of single-crystal silicon.

In recent years, researchers have further broadened the application scope of ion implantation-assisted cutting. For example, Fan et al. [169] studied the effects of implantation doses on the cutting performance of silicon carbide. Their results indicated that ion implantation significantly improved the machinability of silicon carbide, which is similar to the results from Liu et al. [170] and Tanaka et al. [171]. They also reported that even in the brittle mode, microcrack propagation is suppressed at the interface between the amorphous layer and the crystalline substrate after ion implantation. Using an in-situ scanning electron microscopy observation setup, Xu et al. [172] confirmed the simulation results and found that implantation-induced damage helps to enhance the material’s plastic-forming capability during the machining of brittle materials. To enhance the performance of implantation, Fan et al. [173] proposed a dual-ion implantation method that, after high-temperature annealing, can achieve a higher color-center yield within a depth of 5 nm than that obtained with helium-ion implantation alone.

In practical machining, the thickness of modified layers needs to be greater than the height of the structures to be fabricated. However, with a single ion-beam irradiation process, achieving a thick modified layer requires extremely high ion doses and long implantation times. To maximize the thickness of the modified layer, Wang et al. [174] proposed a technique that combines multiple implantations with diamond cutting in 2019, introducing sufficient lattice damage to improve the machinability of single-crystal Si. This method can significantly reduce the required ion dose, offers substantial flexibility in process-parameter design, and effectively improves machining efficiency. Their results showed that the ductile–brittle transition depth of single-crystal silicon increased from 39.7 nm to 472 nm, and high-quality micropillar arrays were successfully fabricated in the ductile mode via fly cutting. Furthermore, Tan et al. [175] applied this idea to single-crystal germanium and increased the maximum implantation depth of copper ions to 1.6 μm. After implantation, the ductile–brittle transition depth of germanium exceeded 200 nm, and the modified layer effectively suppressed subsurface damage during cutting of the ion-implanted germanium samples, ultimately enabling the successful fabrication of crack-free microlens arrays.

### 3.5. Multi-Field-Assisted Cutting

**Figure 17:**
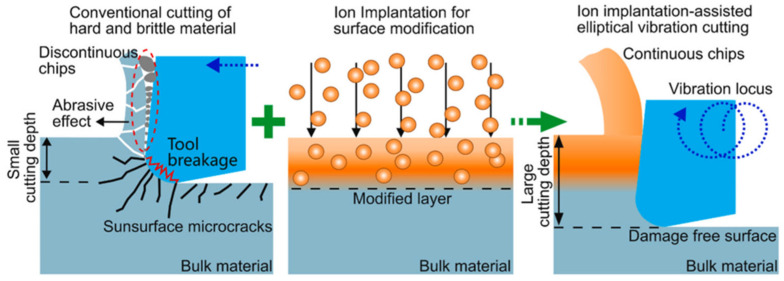
Schematic diagram of multi-field-assisted cutting, which combines ion implantation with tool elliptical vibration [92].

Although the machining performance of brittle materials can be significantly improved by field-assisted cutting, a single field has drawbacks in treating difficult-to-cut materials with specific properties such as extremely high hardness or multiple phases. In recent years, multi-field-assisted cutting technologies have been developed to address the bottlenecks of single fields in UPM [176]. Wang et al. [177] conducted a fundamental study on the combined effects of ion implantation and EVC in the cutting of binderless tungsten carbide. They found that elliptical vibration negatively affects the machinability of superhard materials, whereas ion implantation induces amorphization in the near-surface layer, reducing the strength and brittleness of tungsten carbide. This promotes chip formation via shear deformation rather than brittle fracture and effectively suppresses tool wear. Ke et al. [92] further used this method in ultra-precision cutting of sapphire. They used high-energy ions to modify the workpiece surface before cutting, as shown in Figure 17. Their results indicate a great enhancement in the machinability of sapphire by combining ion implantation with tool vibration. While Zhang et al. [178] coupled LAC and EVC to fabricate silicon microlens arrays. Their results suggest that the combination of the single assistive fields shows great advantages for the fast fabrication of microstructure arrays on brittle materials.

In multi-field-assisted cutting, the material deformation behavior is more complex than that in conventional cutting and single-field-assisted cutting, as the coupling effect of the assistive fields involves sophisticated interactions between workpieces and tools. For example, Liu et al. [179] investigated the coupled effect of LAC and EVC on the material removal mechanism of single-crystal silicon. Their results indicate that the thermal softening effect and the variation in material deformation introduced by tool vibration result in a significant improvement in the machinability of brittle materials. However, the upward motion driven by tool vibration causes undesirable rolling of detached grains, which is more apparent under high-temperature conditions. These findings demonstrated the feasibility of multi-field assistance in improving the machinability of brittle materials and the coupled effect of multi fields on material removal behavior. However, the material properties and deformation behavior under multiple fields are complex, and optimization of the machining process requires the coupling of more field parameters. Therefore, further simulation and experimental investigations are needed to fully reveal the machining mechanism and broaden the application of multi-field-assisted cutting.

## 4. Summary

With the rising demand for precision instruments in optics and aerospace, the demand for ultra-precision machining of brittle materials has continued to grow. This paper summarized the brittle materials commonly used in optics, aerospace, and related fields, as well as their deformation and removal mechanisms during ultra-precision cutting. Then, this paper reviewed the development of field-assisted cutting technologies including laser, vibration, magnetic field, and ion implantation, and discussed the material removal mechanisms involved in these processes. The key conclusions are summarized as follows:

(1). This review surveyed the previous research on the machining mechanism of brittle materials during SPDT and synthesized numerical and experimental findings across material classes including single crystals, polycrystalline ceramics and amorphous glass. Because conventional ultra-precision machining faces difficulty in improving machining ability, efficiency, and stability, the review focuses on the effect of laser heating, tool vibration, magnetic field, and ion implantation on broadening the window of ductile-regime machining of brittle materials.

(2). The main field-assisted cutting approaches reviewed are laser-assisted cutting, vibration-assisted cutting, magnetic field-assisted cutting, and ion implantation-assisted cutting. The suitable materials, processing characteristics, advantages and disadvantages of these techniques are summarized in Table 2.

(3). In addition to single field-assisted cutting, this review highlights the emerging direction of multi-field coupling, noting that combined strategies can outperform single-field assistance. The paper discussed the present multi-field coupling-assisted machining strategies and challenges in understanding the mechanisms of multi-field-assisted cutting. The machining mechanism during multi-field-assisted machining is more complex, involving deformation under various thermal, mechanical, and magnetic conditions.

(4). During machining, the selection of the energy field requires comprehensive consideration of material properties, processing costs and applications. The selection of energy fields should be based on the problems encountered in processing, with a comprehensive consideration of the hybrid effects and equipment construction. The summary and review provide ideas and references for research in solving the problems during ultra-precision cutting of brittle materials and exploration of the mechanisms of field-assisted cutting.

## 5. Future Research Perspectives

(1). Simulation across scales can be effective in the exploration of mechanisms of field-assisted machining. MD simulation can capture phase transitions, amorphization, and dislocation activity in nanometric cutting, but typical domain/time scales limit realistic EVC trajectories and surface formation. Future work can be conducted by coupling MD with the FE method for LAC and EVC, using DEM/phase-field + MD for polycrystalline ceramics where grain boundaries dominate cracking, and validating multiscale models with in-situ measurements during the machining process.

(2). The development of models that predict tool wear, subsurface damage and critical material removal thickness by explicitly coupling tool geometry, workpiece material, and field variables (temperature in LAM, vibration kinematics in EVC, implantation damage gradients). Current indicators such as RTS are useful in predicting material removal mechanisms, but future work should draw more attention to predicting tool wear, subsurface damage and critical material removal thickness in coupling with field variables that reflect thermo-mechanical softening, intermittent contact, and features of modified surface layers.

(3). Multi-field coupling mechanisms (LAC–VAC, implantation–VAC, magnetic–VAC) and synergy/antagonism maps. The review notes that coupled-field effects remain unclear. Future studies should build synergy maps that indicate when a second field enhances or undermines the first. This requires controlled experiments and simulations that isolate coupling terms such as temperature-dependent friction reversal, vibration-modulated heat flux, and substrate interface shielding.

(4). Field-assisted cutting is a promising method to improve the machining performance of various brittle materials. While the distinction between laboratory-scale techniques and industrially mature processes is obvious, laboratory-scale field-assisted cutting techniques often involve custom-built experimental setups, controlled conditions, and small-scale samples, which often face limitations such as complex equipment requirements, difficulty in maintaining process stability, and challenges in integrating with standard CNC systems for reliable operation, repeatable results, and economic viability for large-scale production. Therefore, developing industrially mature processes is critical for the application of field-assisted cutting.

## Figures and Tables

**Table 1:** Comparison between conventional cutting and laser-assisted cutting of several materials (conventional/laser-assisted cutting).

Material	Machined Surface Roughness (nm)	Critical Depth of Cut (nm)
Silicon	Sa: 6.006/2.630 [103]	150/395 [108]
Germanium	Ra: 12.756/9.898 [109]	42.095/211.286 [109]
Magnesium fluoride	Sa:21.289/3.911 [110]	555.23/1079.30 [110]
Fused silica	Sa: 113.362.714 [111]	35.519/112.638 [111]

**Table 2:** Comparison between field-assisted cutting technologies.

Assistive Field	Suitable Materials	Advantages	Disadvantages
Laser-assisted cutting	Single-crystal silicon carbide; fused silica; tungsten carbide; single-crystal silicon	Lower cutting forces; improved machinability; higher material removal rates; better surface finish; lower subsurface damage	Introducing thermal expansion and damage; risking tool softening and chemical wear
Vibration-assisted cutting	Stainless steel; calcium fluoride; single-crystal silicon; tungsten alloy	Lower cutting forces; better surface finish; reduced tool wear; higher accuracy in microstructured surface	Introducing complex parameter-dependent surface-generation behavior; reducing machining efficiency
Magnetic field-assisted cutting	Titanium alloy; calcium fluoride	Decreasing the temperature in the deformed region; suppressing the vibration of tool holder; reducing built-up edge and adhesion	Limited suitable materials; magnetization of workpiece material
Ion implantation-assisted cutting	Single-crystal silicon; single-crystal silicon carbide; sapphire	Lower cutting forces; improved surface finish; improved machinability	High cost; low efficiency; potential for introducing undesired elements into the workpiece

## Data Availability

The original contributions presented in this study are included in the article. Further inquiries can be directed to the corresponding authors.
